# Association of Plasma Epstein-Barr Virus DNA With Outcomes for Patients With Recurrent or Metastatic Nasopharyngeal Carcinoma Receiving Anti–Programmed Cell Death 1 Immunotherapy

**DOI:** 10.1001/jamanetworkopen.2022.0587

**Published:** 2022-03-01

**Authors:** Jian-Ying Xu, Xiao-Li Wei, Chao Ren, Yang Zhang, Yao-Fang Hu, Jia-Yu Li, Jun-Liang Chen, Yi-Qin Wang, Fei Han, Feng-Hua Wang

**Affiliations:** 1 Department of Medical Oncology, Sun Yat-sen University Cancer Center, State Key Laboratory of Oncology in South China, Collaborative Innovation Center for Cancer Medicine, Guangzhou, China; 2 Department of Clinical Research, Sun Yat-sen University Cancer Center, State Key Laboratory of Oncology in South China, Collaborative Innovation Center for Cancer Medicine, Guangzhou, China; 3 Department of Oncology, the Air Force Hospital of Southern Theater Command, Guangzhou, China; 4 Department of Clinical Medicine, Sun Yat-sen University, Guangzhou, China; 5 Department of Radiation Oncology, Sun Yat-sen University Cancer Center, State Key Laboratory of Oncology in South China, Collaborative Innovation Center for Cancer Medicine, Guangzhou, China

## Abstract

**Importance:**

Anti–programmed cell death 1 (anti–PD-1) immunotherapy features a durable response and improved survival in a small subset of patients with recurrent or metastatic nasopharyngeal carcinoma (RM-NPC). The association between plasma Epstein-Barr virus (EBV) DNA titer dynamics and efficacy of anti–PD-1 monotherapy has been reported, while its value in predicting long-term outcomes and monitoring disease progression is unclear for patients with RM-NPC who are receiving anti–PD-1 monotherapy.

**Objective:**

To evaluate the role of plasma EBV DNA titers in prognosis prediction and surveillance of disease progression for patients with RM-NPC who are receiving anti–PD-1 monotherapy.

**Design, Setting, and Participants:**

Patients with RM-NPC from the POLARIS-02 prospective clinical trial, the largest cohort to receive anti–PD-1 monotherapy, were included in this study. From December 22, 2016, to February 19, 2019, 17 participating centers in China screened 279 patients with RM-NPC; 190 patients were enrolled and followed up until February 19, 2020. Plasma EBV DNA was detected before treatment and every 4 weeks until disease progression.

**Main Outcomes and Measures:**

Plasma EBV DNA as a predictor for progression-free survival (PFS), overall survival (OS), durable clinical benefit (defined as PFS of ≥6 months), and disease progression.

**Results:**

Of 179 patients with RM-NPC receiving anti–PD-1 therapy, 148 (82.7%) were men, and the median age was 46 years (range, 22-71 years). A higher baseline EBV DNA titer was associated with shorter median OS (hazard ratio, 1.88; 95% CI, 1.22-2.89; *P* = .004). Patients with a ratio of the EBV DNA titer at week 4 to that at baseline (W4 to baseline ratio) greater than 0.5 had shorter median OS (hazard ratio, 2.18; 95% CI, 1.30-3.65; *P* < .001) than those with a W4 to baseline ratio of 0.5 or less. Patients with higher baseline EBV DNA titers had a lower durable clinical benefit rate than those with lower baseline EBV DNA titers (19 of 97 [19.6%] vs 27 of 71 [38.0%]; *P* = .01). Similarly, patients with a W4 to baseline ratio greater than 0.5 had a lower durable clinical benefit rate than those with a W4 to baseline ratio of 0.5 or less (9 of 86 [10.5%] vs 32 of 54 [59.3%]; *P* < .001). In addition, a significant EBV DNA titer increase was present at a median of 2.6 months (IQR, 0.9-4.5 months) prior to radiographic progression.

**Conclusions and Relevance:**

This study of plasma EBV DNA in patients with RM-NPC who are receiving anti-PD-1 monotherapy suggests that plasma EBV DNA could be a useful biomarker for outcomes and monitoring disease progression.

## Key Points

**Question:**

Are plasma EBV DNA titers associated with outcomes for patients with recurrent or metastatic nasopharyngeal carcinoma (RM-NPC) receiving anti–programmed cell death 1 (anti–PD-1) monotherapy?

**Findings:**

In this prognostic study of 190 patients with RM-NPC receiving anti–PD-1 monotherapy, baseline plasma EBV DNA titers and their dynamics were significantly associated with progression-free survival, overall survival, and durable clinical benefit (defined as progression-free survival of ≥6 months). A significant plasma EBV DNA titer increase occurred prior to radiographic disease progression.

**Meaning:**

This study suggests that longitudinal plasma EBV DNA monitoring could be incorporated into the guidance of personalized disease management and future clinical trials for patients with NPC in the era of immunotherapy.

## Teaser

This prognostic study evaluates the role of plasma Epstein-Barr virus (EBV) DNA titers in prognosis prediction and surveillance of disease progression for patients with recurrent or metastatic nasopharyngeal carcinoma who are receiving anti–programmed cell death 1 (anti–PD-1) monotherapy.

## Introduction

Nasopharyngeal carcinoma (NPC) has a distinct geographic distribution, with a predominance in Asia and North Africa.^1^ The prognosis of patients with recurrent or metastatic NPC (RM-NPC) who fail to respond to standard chemotherapy is poor.^2^ There had been no effective treatment until anti–programmed cell death 1 (anti–PD-1) therapy achieved breakthroughs in previously treated patients with RM-NPC. Although the objective response rates (ORRs) range from only 20.5% to 34.0%, the improvements in the the median duration of response and in overall survival (OS) are notable.^3,4,5^ However, there is a lack of effective biomarkers to identify patients who are likely to gain long-lasting clinical benefits and to allow for appropriate immunotherapy tailoring for patient subgroups.^6,7,8^

Nasopharyngeal carcinoma has a close association with Epstein-Barr virus (EBV).^1^ Plasma EBV DNA has been established as an effective marker for chemotherapy and radiotherapy.^6,7,8,9,10,11^ Anti–PD-1 therapy has a unique mechanism and therapeutic characteristics that are distinct from those of chemotherapy and radiotherapy.^12^ The value of plasma EBV DNA as a biomarker in the era of NPC immunotherapy needs to be verified. A phase 2 study (NCI-9742) of nivolumab in RM-NPC showed that there were no statistical differences in the ORR or survival between patients with plasma EBV DNA clearance above or below the median half-life or between patients with increasing EBV DNA titers and patients with decreasing EBV DNA titers during the first month of treatment.^3^ The POLARIS-02 study, a larger study evaluating the efficacy and safety of toripalimab in chemorefractory RM-NPC, showed that patients with pretreatment EBV DNA titers less than 10 000 IU/mL did not have a significantly higher ORR than those with EBV DNA titers of 10 000 IU/mL or more, while patients with a 50% or greater decrease in plasma EBV DNA titers on day 28 had a significantly better ORR than those with a decrease of less than 50%.^13^ Uncertainty exists around the associations of plasma EBV DNA with long-term outcomes of NPC immunotherapy given the limited clinical evidence. However, the associations between plasma EBV DNA and survival, durable clinical benefit (DCB), and disease progression remain unclear for patients with RM-NPC who are receiving anti–PD-1 monotherapy. Hence, we analyzed the data of patients with RM-NPC from a prospective clinical study (POLARIS-02) with the largest cohort to date of patients with RM-NPC treated with anti–PD-1 monotherapy, and we aimed to explore the role that plasma EBV DNA titers and their dynamics play in prognosis prediction and surveillance of disease progression for patients with RM-NPC who are receiving anti–PD-1 monotherapy in the era of immunotherapy.

## Methods

### Patients and Data Collection

The design and results of the POLARIS-02 phase 2 multicenter clinical trial evaluating toripalimab for patients with RM-NPC (ClinicalTrials.gov identifier: NCT02915432) have been previously published.^13^ Our prognostic study was approved by the institutional ethics committee of Sun Yat-sen University Cancer Center and was conducted in accordance with the Declaration of Helsinki.^14^ Written informed consent was obtained from study participants. This study followed the Transparent Reporting of a Multivariable Prediction Model for Individual Prognosis or Diagnosis (TRIPOD) reporting guideline.

From December 22, 2016, to February 19, 2019, 17 participating centers in China screened 279 patients with RM-NPC; 190 patients were enrolled and followed up until February 19, 2020. Patients with RM-NPC received toripalimab, 3 mg/kg, once every 2 weeks. Tumor response assessments were performed every 8 weeks during the first year of treatment and then every 12 weeks until disease progression or therapy discontinuation. Data on clinical characteristics, plasma EBV DNA titers, tumor programmed death ligand 1 expression, tumor mutation burden, response, and survival outcome were collected. Patients without baseline EBV DNA results were excluded from this study.

### Sample Collection and Analysis

The plasma EBV DNA copy number was determined in the central laboratory by real-time quantitative polymerase chain reaction with probes against EBV genes 1 day before treatment and every 4 weeks until disease progression. Real-time quantitative polymerase chain reaction was performed with the ABI Prism 7500 Sequence Detection Analyzer (Applied Biosystems). The agents for the DNA extraction and detection kit were commercially available tests supplied by Sansure Biotech. The DNA extraction and detection kit has been approved by the National Medical Products Administration and Conformité Européenne. Quantitative performance validation was qualified, including the following parameters: trueness, precision (precision of repeatability and intermediate precision), linearity verification, limit of detection, and analytical specificity.

The baseline EBV DNA titer was identified with a cutoff value of 10 000 IU/mL.^13^ The W4 to baseline ratio was defined as the ratio of the EBV DNA titer at week 4 to that at baseline. The EBV DNA titer of each patient at baseline, week 4, and week 8 was plotted. A line graph of the EBV DNA titer change was used to classify an increasing trend and a decreasing trend. An increase of EBV DNA titer was considered significant if if the fold increase was more than 50% or 2 successive increases were observed during treatment.

### Statistical Analysis

Antitumor effectiveness was assessed by the independent review committee according to Response Evaluation Criteria in Solid Tumors v1.1.^15^ Progression-free survival (PFS) was calculated as the time from the first treatment to the first recorded instance of progression of disease or death, whichever came first. Durable clinical benefit was defined as a PFS of at least 6 months. No durable benefit was defined as progression of disease or stable disease that lasted 6 months or less.^16^ Patients who had not progressed and were censored before 6 months of follow-up were considered not evaluable and excluded from the analysis of DCB. Overall survival was defined as the duration between the first dose of toripalimab and death due to any cause. Patients with progression identified in the second or later imaging were considered to have secondary progression.

We aimed to explore the value of plasma EBV DNA titers and their dynamics to predict outcomes and disease progression. R, version 3.6.1 (R Group for Statistical Computing) was used to perform our analyses. Cases with missing data were omitted. A 2-tailed *P* < .05 was considered to indicate statistical significance as the power test. Durable clinical benefit rates were compared with the Pearson χ^2^ test. The Mann-Whitney test was used to compare the fold decrease of EBV DNA in patients with DCB or no durable benefit. Survival curves were plotted using the Kaplan-Meier method and compared with the log-rank test. Hazard ratios (HR) and 95% CIs were calculated using the Cox proportional hazards regression model. The proportional hazards assumption was checked using statistical tests and graphical diagnostics based on the scaled Schoenfeld residuals. Both individual tests for variates and the global tests were not statistically significant (all *P* > 0.1), which suggests that we could not reject the assumption of proportional hazards.

## Results

### Patient Cohort

A total of 190 patients were enrolled in the prospective phase 2 clinical trial of anti–PD-1 monotherapy for patients with RM-NPC; 11 patients without data on baseline EBV DNA titers were excluded from this analysis. Thus, a total of 179 patients (148 men [82.7%]; median age, 46 years [range, 22-71 years]) were included for the analysis of baseline EBV DNA titers. A total of 31 patients were excluded (4 patients dropped out because of disease progression, and 27 patients were lost to follow-up, withdrew consent, or had a protocol deviation before the first 4 weeks). Thus, 148 patients had dynamic monitoring of EBV DNA titers during treatment. Patients’ clinical characteristics are shown in the Table. There were no significant associations between baseline EBV DNA titers and clinical characteristics (eTable in the Supplement).

**Table.** Characteristics of Patients

Characteristic	Patients, No. (%) (N = 179)
Sex	
Male	148 (82.7)
Female	31 (17.3)
Age, median (range), y	46 (22-71)
ECOG score	
0	63 (35.2)
1	116 (64.8)
Lines of prior therapy	
1 line	89 (49.7)
≥2 lines	90 (50.3)
PD-L1 status^a^	
Negative	126 (70.4)
Positive	45 (25.1)
NA	8 (4.5)
TMB (mutations per megabase)^b^	
<0.95	82 (45.8)
≥0.95	82 (45.8)
NA	15 (8.4)

Abbreviations: ECOG, Eastern Cooperative Oncology Group; NA, not applicable; PD-L1, programmed death-ligand 1; TMB, tumor mutation burden.

^a^ Positive is defined as 1% or more of tumor cells expressing PD-L1 by SP142 immunohistochemistry staining.

^b^ Cutoff value is defined as the median value of TMB.

### Association Between EBV DNA Titers and Survival

**Figure 1.**
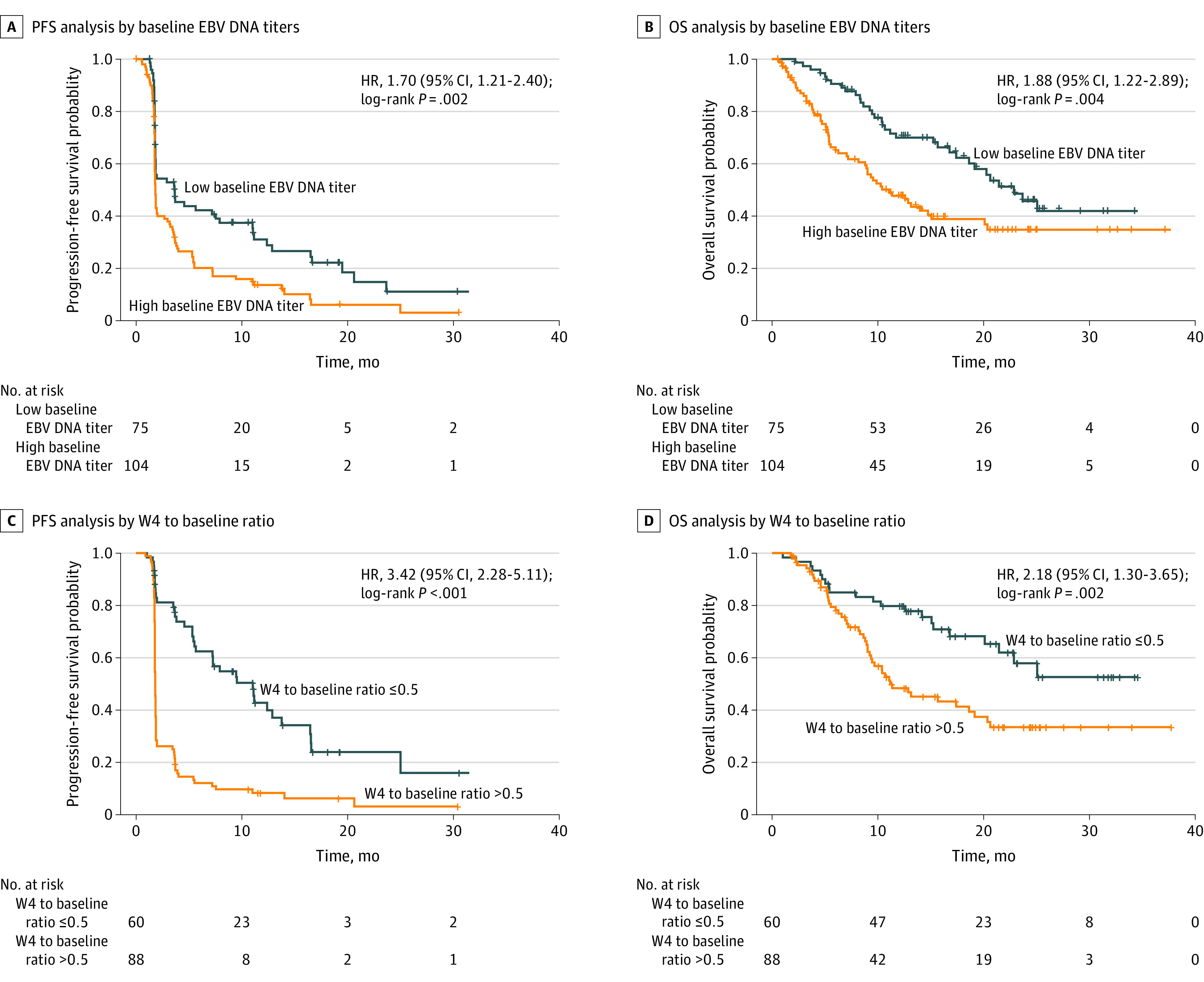
Progression-Free Survival (PFS) Analysis and Overall Survival (OS) Analysis A, PFS analysis of different baseline Epstein-Barr virus (EBV) DNA titer groups. B, OS analysis of different baseline EBV DNA titer groups. C, PFS for patients with a ratio of the EBV DNA titer at week 4 to that at baseline (W4 to baseline ratio) of 0.5 or less and patients with a W4 to baseline ratio of more than 0.5. D, OS for patients with a W4 to baseline ratio of 0.5 or less and patients with a W4 to baseline ratio of more than 0.5. The cutoff value for the baseline EBV DNA titer was 10 000 IU/mL.

The plasma EBV DNA titer at baseline was detectable for 179 patients. With a cutoff value of 10 000 IU/mL,^12^ the patients were divided into the low baseline EBV DNA group (<10 000 IU/mL; n = 75) and the high baseline EBV DNA group (≥10 000 IU/mL; n = 104). Patients in the high baseline EBV DNA group had significantly shorter median PFS than those in the low baseline EBV DNA group (1.8 months [IQR, 1.8-2.7 months] vs 3.7 months [IQR, 1.9-11.0 months]; HR, 1.70; 95% CI, 1.21-2.40; *P* = .002) (Figure 1A), as well as significantly shorter median OS (11.1 months [IQR, 8.9-20.1 months] vs 22.9 months [IQR, 18.6 months to unreached]; HR, 1.88; 95% CI, 1.22-2.89; *P* = .004) (Figure 1B).

Dynamic monitoring of the plasma EBV DNA titers was performed, and data from 148 patients were available. Patients were divided into 2 groups according to the ratio of W4 to baseline: 0.5 or less (n = 60) and more than 0.5 (n = 88). Patients with a W4 to baseline ratio of more than 0.5 had shorter median PFS than those with a W4 to baseline ratio of 0.5 or less (1.8 months [IQR, 1.7-1.8 months] vs 11.0 months [IQR, 7.2-16.5 months]; HR, 3.42; 95% CI, 2.28-5.11; *P* < .001) (Figure 1C), as well as shorter median OS (11.2 months [IQR, 9.3-20.4 months] vs unreached [IQR, 21.5 months to unreached]; HR, 2.18; 95% CI, 1.30-3.65; *P* = .002) (Figure 1D). Our results suggest that the plasma EBV DNA titer at baseline and the W4 to baseline ratio can be used as valuable prognostic markers.

### Association Between EBV DNA Titers and DCB

**Figure 2.**
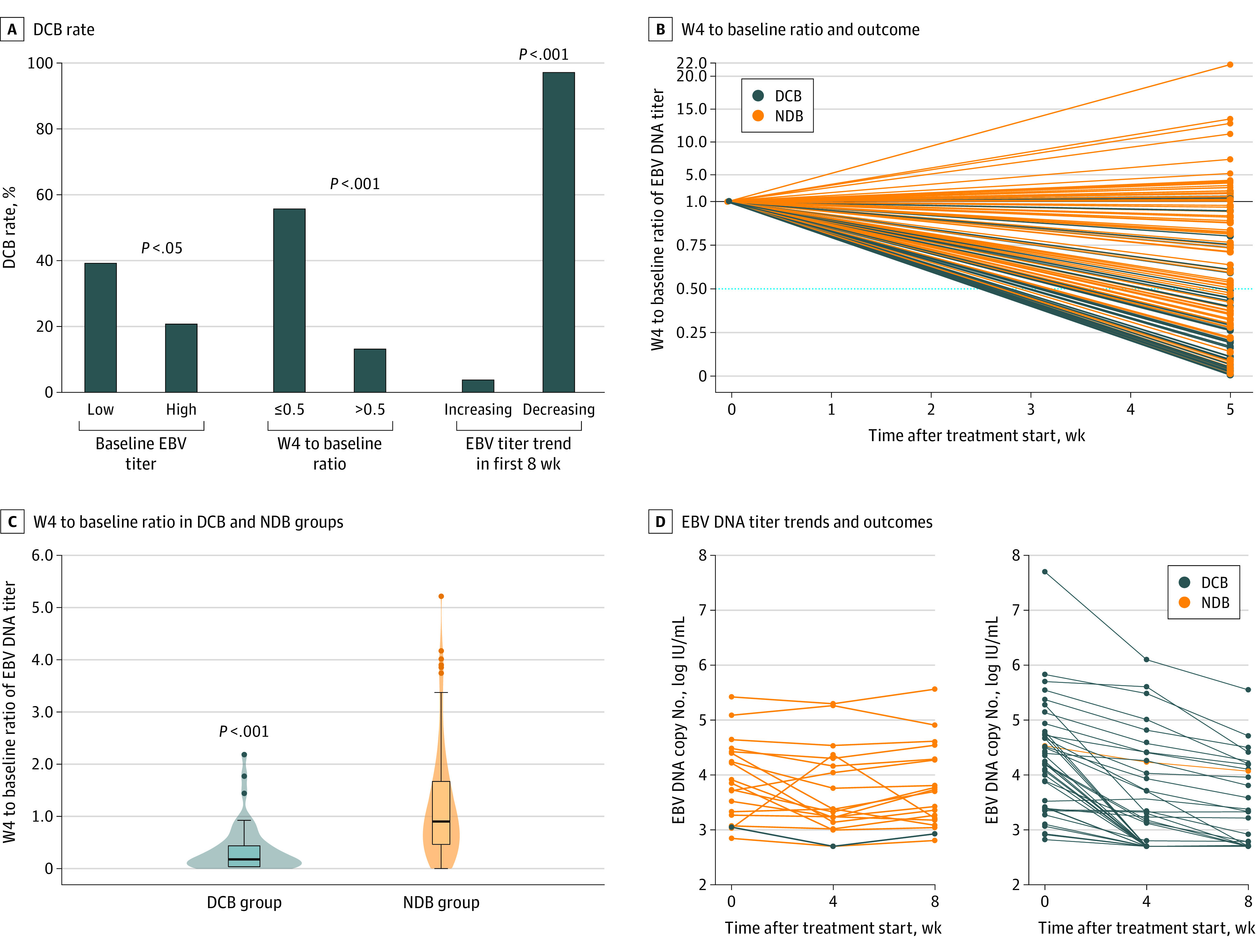
Association Between Epstein-Barr Virus (EBV) DNA Titer and an Ultimate Outcome of Durable Clinical Benefit (DCB) A, DCB rate for patients with different baseline EBV DNA titers (left), a ratio of the EBV DNA titer at week 4 to that at baseline (W4 to baseline ratio) (middle), and the trend of EBV DNA titer during the first 8 weeks (right). B, W4 to baseline ratio of EBV DNA titer and the outcome of DCB or no durable benefit (NDB). The black horizontal line at 1.0 and the blue horizontal dotted line at 0.50 indicate a W4 to baseline ratio of 0.50 and 1.0. C, W4 to baseline ratio of EBV DNA titer in the DCB group and NDB group. The dots indicate the ratio. To clearly show the results, we omitted 5 outliers (7.4, 11.2, 12.8, 13.5, and 21.8 in the NDB group). D, Patients with an increasing trend (left) or decreasing trend (right) of EBV DNA titer during the first 8 weeks and their ultimate outcomes (DCB or NDB). The cutoff value for the baseline EBV DNA titer was 10 000 IU/mL. Durable clinical benefit was defined as progression-free survival of at least 6 months. No durable benefit was defined as progression of disease or stable disease that lasted 6 months or less. The fold decrease was defined as the W4 to baseline ratio. A decreasing trend was defined as no increase in EBV DNA titer during the first 8 weeks of therapy; if patients did not meet this requirement, they were said to have an increasing trend.

The DCB rate is recommended as a surrogate end point of long-term clinical benefit from immune checkpoint inhibitors.^17^ We further investigated the association between baseline and dynamic EBV DNA titers and DCB. Of 179 patients, 46 (25.7%) obtained DCB, and 122 (68.2%) had no durable benefit, while 11 (6.1%) were not evaluable. Patients in the low baseline EBV DNA group (n = 71) achieved a significantly higher DCB rate than those in the high baseline EBV DNA group (n = 97) (27 of 71 [38.0%] vs 19 of 97 [19.6%]; *P* < .05) (Figure 2A).

We explored the association between the W4 to baseline ratio and DCB. Of the 148 patients with dynamic EBV DNA titer results, 8 were not evaluable. A total of 140 patients were divided into the group with a W4 to baseline ratio of 0.5 or less (n = 54) or the group with a W4 to baseline ratio of more than 0.5 (n = 86). Of these, 32 of 54 patients (59.3%) with a W4 to baseline ratio of 0.5 or less obtained DCB, while 9 of 86 patients (10.5%) with a W4 to baseline ratio of more than 0.5 obtained DCB (eFigure, A, in the Supplement; *P* < .001). Most patients with DCB had a W4 to baseline ratio of 0.5 or less (Figure 2B). The median W4 to baseline ratio for the patients with DCB was lower than that for those without DCB (0.20 [IQR, 0.03-0.40] vs 1.02 [IQR, 0.53-1.68]; *P* < .001 (Figure 2C).

We further explored the association between the trend of EBV DNA titers during the first 8 weeks and DCB. Of 148 patients with dynamic EBV DNA titer results, 88 were excluded (80 patients had progressed by the first evaluation, and 8 patients were not evaluable). We plotted EBV DNA titer changes of the remaining 60 patients in line graphs and divided the patients into an increasing trend group (n = 19) and a decreasing trend group (n = 41) (Figure 2D). Almost all of the patients (40 of 41 [97.6%]) in the decreasing trend group ultimately obtained DCB, while only 1 patient (1 of 19 [5.3%]; *P* < .001) in the increasing trend group obtained DCB (Figure 2A). Therefore, the trend of EBV DNA titers during the first 8 weeks could be used to identify patients with DCB.

### Association Between EBV DNA Titers and Secondary Disease Progression

We explored the value of EBV DNA titer dynamic changes in predicting secondary disease progression. Of 148 patients with dynamic EBV DNA titer results, 87 were excluded (80 patients had progressed by the first evaluation, and 7 patients did not have complete dynamic EBV DNA titer results). We plotted a line graph of the EBV DNA titers of the remaining 61 patients. The EBV DNA titer dynamic change trends of 30 patients with complete response or partial response are shown in eFigure, A, in the Supplement. We found that 18 patients without any significant increase in EBV DNA titers as indicated in Methods were free from disease progression, while 12 patients with significant increases in EBV DNA titers during treatment subsequently developed disease progression. eFigure, B, in the Supplement shows the EBV DNA titer dynamic change trends of 31 patients with stable disease. All of them experienced significant increases in EBV DNA titers during treatment. Of these 31 patients, 23 (74.2%) subsequently developed disease progression, and 8 (25.8%) did not have disease progression.

**Figure 3.**
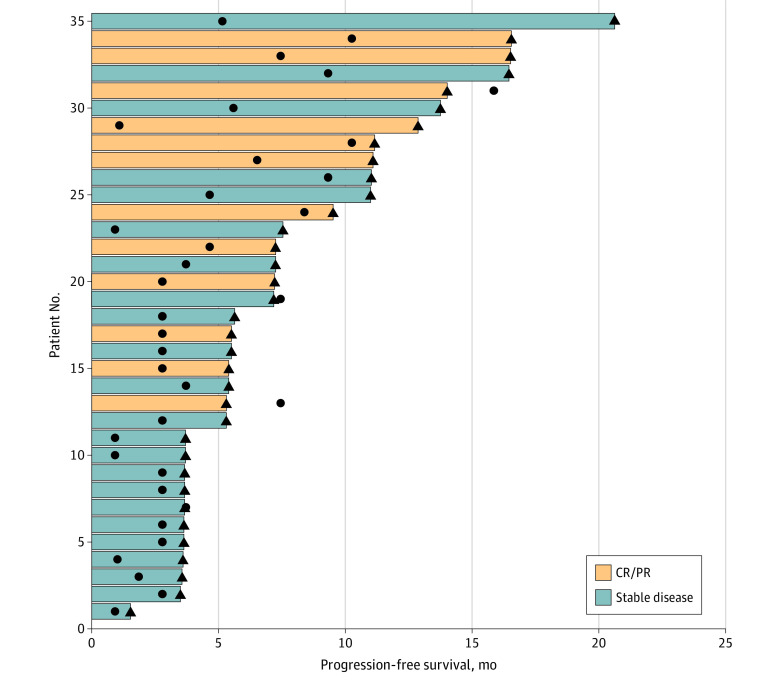
Swimmer Plot for 35 Patients With Complete Response or Partial Response (CP/PR) or Stable Disease Triangles indicate the time of disease progression, and circles indicate the occurrence of a significant Epstein-Barr virus DNA titer increase.

Next, we focused on 35 patients who achieved complete response, partial response, or stable disease but later experienced disease progression. These patients had significant increases in EBV DNA titers at a median of 2.6 months (IQR, 0.9-4.5 months) prior to radiographic disease progression (Figure 3). This finding indicates that a significant increase in EBV DNA titers during treatment could predict disease progression early.

## Discussion

With the approval of anti–PD-1 antibodies for different tumor indications, the era of immunotherapy for NPC is coming. To date, the data on the association between the outcomes of anti–PD-1 therapy and plasma EBV DNA levels in patients with RM-NPC are limited. Here, we comprehensively investigated the value of plasma EBV DNA titers and their dynamics as biomarkers for predicting long-term outcomes and monitoring disease progression by analyzing the data from a prospective clinical study with the largest cohort to date of patients with RM-NPC treated with anti–PD-1 monotherapy. We found that patients with a high baseline EBV DNA titer or with a W4 to baseline ratio of more than 0.5 had a significantly shorter median PFS, shorter median OS, and lower DCB rate, which demonstrated that the baseline plasma EBV DNA titer and the degree of EBV DNA titer early decrease can be used as valuable biomarkers for predicting long-term outcomes for patients with RM-NPC receiving immunotherapy.

The controversies regarding the role of plasma EBV DNA titers as a biomarker for patients with RM-NPC treated with anti–PD-1 antibodies remain unresolved owing to the limited data. The Mayo Clinic Phase 2 Consortium study included 44 patients with RM-NPC treated with nivolumab and found no significant differences in survival between the group with an increasing trend in EBV DNA titers and the group with a decreasing trend in EBV DNA titers.^3^ However, a decreasing trend in EBV titers was shown in 87.5% of the responders (7 of 8). The lack of a significant difference may have been associated with the inadequate sample size. In our study, we found improved survival among patients with RM-NPC with low baseline EBV DNA titers and early decreases in EBV DNA titers. The result of the CAPTAIN-1st trial (a study of the addition of camrelizumab to gemcitabine and cisplatin) demonstrated that the early clearance of EBV DNA is associated with the response rate of camrelizumab in combination with gemcitabine and cisplatin.^18^ The first report of the POLARIS-02 study showed that there was no statistically significant association between the baseline EBV titers and ORR.^13^ However, a positive association of plasma EBV DNA copy number reduction with improved response to anti–PD-1 monotherapy was observed. Here, we found improved survival among patients with RM-NPC with low baseline EBV DNA titers and early decreases in EBV DNA titers. Many factors, such as different cutoff levels, analysis method, detection time, or geographic population selection, may be associated with the inconsistency of the published results. More large prospective studies in progress may help provide the necessary conclusions.

The benefit of immunotherapy is its potential for achieving long-lasting clinical benefit and improved long-term survival in a subset of patients.^19^ In our study, the ORR could not successfully identify the patients who obtained long-lasting clinical benefit. Similarly, Nabet et al^20^ analyzed the data from 273 patients with non–small cell lung cancer receiving PD-1 antibody therapy and found that the initial response assessment by conventional imaging could not fully identify which patients could achieve DCB. Ritchie et al^17^ analyzed 87 phase 2 trials and indicated that ORR was poorly associated with OS and that only the DCB rate could be recommended as an end point for long-term survival. However, how to identify patients with RM-NPC who are likely to achieve DCB remains unclear. To our knowledge, we are the first to explore DCB for patients with RM-NPC and identify those patients with a higher baseline EBV DNA titer or a W4 to baseline ratio of more than 0.5 who were associated with a lower DCB rate. A model for predicting DCB could be established for anti–PD-1 immunotherapy in RM-NPC by combining EBV DNA parameters and other factors, which could further aid patient selection.

Serial testing of EBV DNA is considered to be a useful and noninvasive method for monitoring tumor response during neoadjuvant chemoradiotherapy.^2^ However, the value of EBV surveillance for patients with RM-NPC receiving anti–PD-1 immunotherapy is unclear. We investigated the association between an increase in EBV DNA and disease progression. No significant increase in EBV DNA titer was observed in patients without disease progression. Half of these patients achieved a long median PFS of 12 months. An increase in EBV DNA titers was shown in all patients, with disease progression approximately 2.6 months prior to radiologic review. Our results demonstrate the role of plasma EBV DNA monitoring during immunotherapy, which aids monitoring and enables adjustment of therapy in a timely manner. A possible underlying mechanism for this phenomenon could be tumor evasion from the immune system. The EBV-encoded latent membrane proteins limit the actions of interferon by targeting interferon receptors for degradation, which allows NPC cells to avoid immune surveillance.^21^ In addition, EBV noncoding RNA molecules enable NPC cells to escape immune recognition by blocking antigen presentation and immune cell activation.^22,23^ Therefore, a heavy load or an increase in EBV DNA could be associated with more tumor cells escaping immune destruction and result in a poor prognosis and disease progression.

### Limitations

Some limitations existed in this study. First, the risk of patient selection bias was inevitable because the data were based on clinical trials, suggesting that our results may not completely represent all real-world patients. Second, the mechanism of plasma EBV DNA predicting the effectiveness and prognosis of immunotherapy is complex, whether there are immune-related factors or manifestations of reduced tumor load. More prospective studies with larger cohorts are needed to support our results.

## Conclusions

This study suggests that baseline plasma EBV DNA levels and their dynamic changes may help identify patients with RM-NPC who are likely to obtain DCB and prolonged survival with anti–PD-1 immunotherapy. Longitudinal EBV DNA measurements may identify patients with disease progression prior to radiologic findings. This study supports the idea that plasma EBV DNA detection and its dynamic monitoring could be incorporated into the guidance of personalized disease management and future clinical trials of treatment for NPC in the era of immunotherapy.

## Notes

Supplement. **eTable.** The Correlation Between Baseline EBV DNA Titer and Clinical Factors **eFigure.** Association of a Dynamic Change in EBV DNA Titer and Disease Status (Progression or Free From Progression)
