# Supplementary material for: Association of Plasma Epstein-Barr Virus DNA With Outcomes for Patients With Recurrent or Metastatic Nasopharyngeal Carcinoma Receiving Anti–Programmed Cell Death 1 Immunotherapy
Source: JAMA Netw Open. 2022 Mar 1;5(3):e220587. doi: 10.1001/jamanetworkopen.2022.0587 (PMC8889459; doi:10.1001/jamanetworkopen.2022.0587)
Supplement: Supplement. — eTable. The Correlation Between Baseline EBV DNA Titer and Clinical Factors eFigure. Association of a Dynamic Change in EBV DNA Titer and Disease Status (Progression or Free From Progression) [file jamanetwopen-e220587-s001.pdf]

Supplementary Online Content

Xu JY, Wei XL, Ren C, et al. Association of plasma Epstein-Barr virus DNA with outcomes for patients with recurrent or metastatic nasopharyngeal carcinoma receiving anti–programmed cell death 1 immunotherapy. *JAMA Netw Open.* 2022;5(3):e220587. doi:10.1001/jamanetworkopen.2022.0587

eTable. The Correlation Between Baseline EBV DNA Titer and Clinical Factors
eFigure. Association of a Dynamic Change in EBV DNA Titer and Disease Status (Progression or Free From Progression)

This supplementary material has been provided by the authors to give readers additional information about their work.

© 2022 Xu JY et al. *JAMA Network Open.*

eTable. The Correlation Between Baseline EBV DNA Titer and Clinical Factors

**Factors**	**EBV titer ≥10,000 IU/mL**	**EBV titer <10,000 IU/mL**	***P* value**
**Sex**			0.2816
Male	89 (91.2%)	59 (80.6%)	
Female	15 (8.8%)	16 (19.3%)	
**Age (years)**			0.9799
<46	49 (47.1%)	36 (48.0%)	
≥46	55 (59.6%)	39 (40.6%)	
**Line of prior therapy**			0.8328
1 line	50 (48.1%)	34 (45.3%)	
2+ lines	54 (51.9%)	41 (54.7%)	
**ECOG**			0.7761
0	38 (36.5%)	25 (15.5%)	
1	66 (63.5%)	50 (66.7%)	
**Histology**			0.9906
Nonkeratinizing	100 (96.2%)	73 (17.2%)	
Keratinizing	4 (3.8%)	2 (2.7%)	
**PD-L1 status** [a]			0.3893
Negative	70 (70.7%)	56 (77.8%)	
Positive	29 (29.3%)	16 (22.2%)	
**TMB (mutation/Mb)** [b]			
TMB < 0.95	45 (46.9)	37 (54.4)	0.9969
TMB≥0.95	51 (53.1)	31 (45.6)	

[a] Positive is defined as ≥1% of tumor cells expressing PD-L1 by SP142 immunohistochemistry staining. [b] The cutoff value is defined as the median value of TMB.

Abbreviations: ECOG, Eastern Cooperative Oncology Group; PD-L1, programmed cell death ligand 1; TMB, tumor mutational burden

© 2022 Xu JY et al. *JAMA Network Open.*

eFigure 1. Association of a Dynamic Change in EBV DNA Titer and Disease Status (Progression or Free From Progression). (A) Association of the EBV DNA titer dynamic change in patients with a best response of complete response or partial response with disease status. (B) Association of the EBV DNA titer dynamic change in patients with a best response of stable disease with disease status.

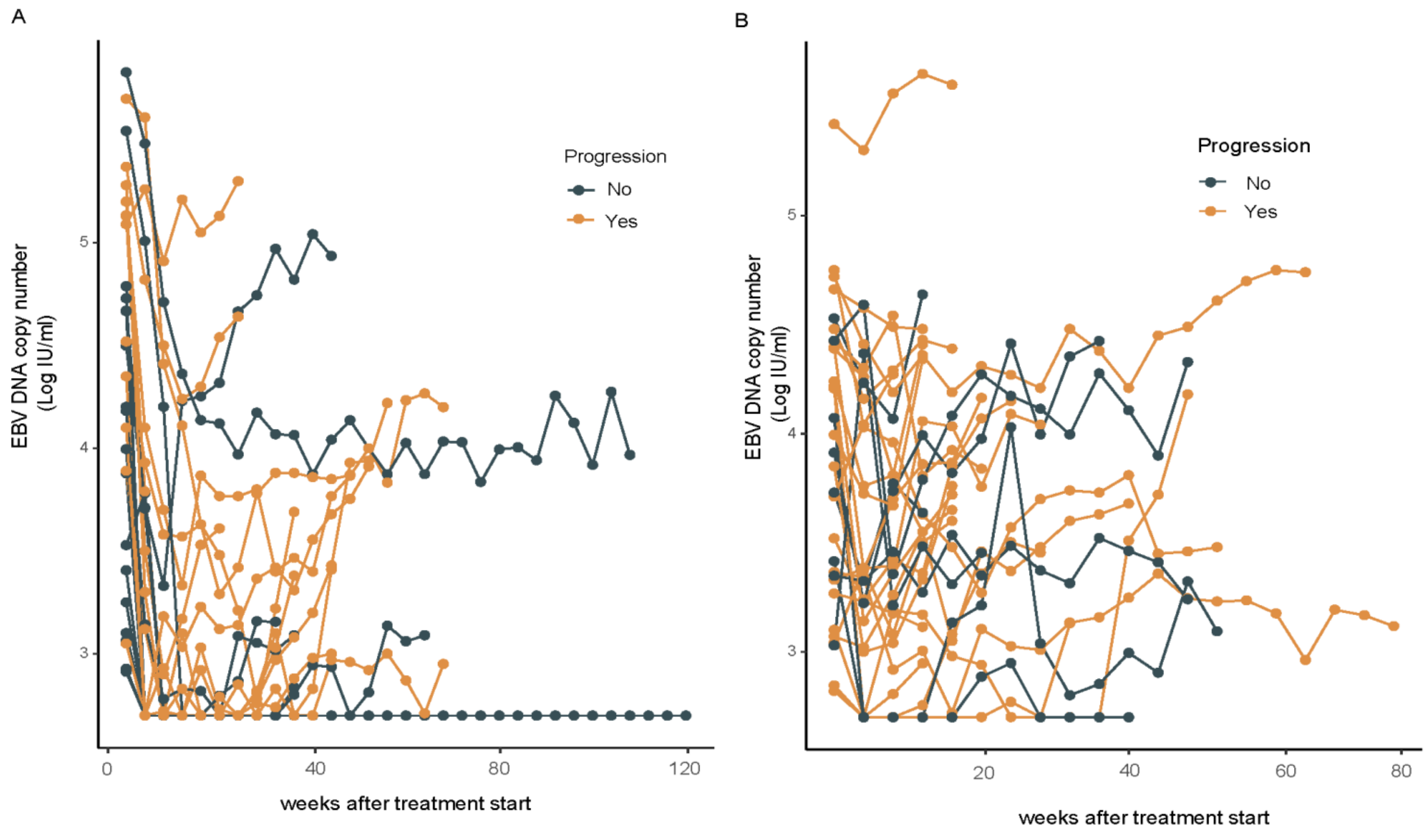

© 2022 Xu JY et al. *JAMA Network Open*.
